# Patient-reported symptoms and burden of eosinophilic esophagitis: evidence from real-world clinical practice

**DOI:** 10.1186/s12876-024-03334-4

**Published:** 2024-08-03

**Authors:** Xiao Xu, Justin Kwiatek, James Siddall, Eduardo Genofre, Heide Stirnadel-Farrant, Rohit Katial

**Affiliations:** 1 BioPharmaceuticals Market Access and Pricing, AstraZeneca, 200 Orchard Ridge Dr, Gaithersburg, MD 20878 USA; 2 BioPharmaceuticals Medical, AstraZeneca, 200 Orchard Ridge Dr, Gaithersburg, MD 20878 USA; 3 Adelphi Real World, Bollington, Cheshire UK; 4 BioPharmaceuticals Medical, AstraZeneca, Wilmington, DE USA; 5 BioPharmaceuticals Medical, AstraZeneca, Cambridge, UK

**Keywords:** Eosinophilic esophagitis, Inflammatory disease, Real-world evidence, Health-related quality of life, Disease burden

## Abstract

**Background:**

Eosinophilic esophagitis is a chronic inflammatory disorder of the esophagus. This real-world study used patient and physician surveys to describe the clinical characteristics and disease burden of eosinophilic esophagitis—overall and in a subgroup of patients with dysphagia despite treatment.

**Methods:**

Data analyzed in this study were collected in 2020 from US and EU patients with eosinophilic esophagitis. Eligible patients were aged ≥ 12 years with a diagnosis of eosinophilic esophagitis, had an esophageal count of ≥ 15 eosinophils/high-power field at diagnosis, and were currently prescribed treatment for eosinophilic esophagitis.

**Results:**

Overall, 1001 patients were included, of whom 356 (36%) had dysphagia despite treatment. Demographics and clinical characteristics were similar in both populations. The severity of eosinophilic esophagitis was mild in more patients overall (69%) versus those with dysphagia despite treatment (48%). Patient disease history was similar in both populations, with some exceptions: common patient-reported symptoms were dysphagia (70% and 86%) and heartburn/acid reflux (55% and 49%), and common physician-reported symptoms were dysphagia (75% and 91%) and food impaction (46% and 52%). Treatment history was similar in both populations; overall, the most common treatments were proton pump inhibitors (83%) and topical corticosteroids (51%). Patients reported slightly more days with symptoms, higher impacts on activities of daily living, and slightly higher anxiety or depression in the dysphagia-despite-treatment population versus the overall population.

**Conclusions:**

Eosinophilic esophagitis presents severe symptoms and comorbidities that substantially impact patients’ well-being and quality of life. Greater awareness of and novel treatments for eosinophilic esophagitis are needed.

**Supplementary Information:**

The online version contains supplementary material available at 10.1186/s12876-024-03334-4.

## Introduction

Eosinophilic esophagitis (EoE) is a chronic, immune-mediated, inflammatory disease of the esophagus [1, 2]. Symptoms of EoE include dysphagia, food impaction, abdominal pain, nausea, and vomiting [1, 2]. Although it does not appear to limit the life expectancy of patients [3], clinical observations indicate that EoE is associated with substantial patient burden and impacts to their health-related quality of life (HRQOL) [4, 5].

The diagnosis of EoE is often performed histologically, with the presence of eosinophils on biopsy, indicating inflammation of esophageal tissue [6]. The current standard of care for EoE often relies on elemental and/or elimination diets and esophageal dilations, in addition to medications, such as proton pump inhibitors and swallowed topical steroids (i.e., budesonide, currently approved in the European Union [EU] [7] and Canada [8] only), to manage the disease [9, 10]. However, patient HRQOL is especially impacted among those with more severe symptoms, histologically active disease, or extensive dietary restrictions [11, 12]; indeed, many patients continue to experience symptoms related to EoE despite current standard-of-care treatments [5]. A few biologic therapies have recently been investigated for EoE, though dupilumab is the only agent in this class currently approved to treat EoE [13]. Although several studies have collected survey data on the patient impact of EoE [3, 5, 11, 12, 14–17], patient-reported evidence is still limited regarding EoE symptoms, EoE disease burden, and EoE impact on patient well-being and activities of daily living. Moreover, social media studies in EoE suggest an unmet patient need for accurate, reliable sources of information about the disease, barriers to treatment, and shared decision-making [18, 19].

The objectives of this real-world study using patient-reported survey responses were to describe the demographics, clinical characteristics, and disease burden of patients with EoE and to locate and highlight any data gaps in patient versus physician perspectives to gain a better understanding of the proper management of patients with EoE.

## Methods

### Study design

This study used real-world data from the Adelphi Real World Disease Specific Programmes™, which are impartial, multinational, cross-sectional physician and patient surveys that provide data related to real-world clinical practices for a range of chronic health conditions, including EoE [20]. The data analyzed in this study were collected from patients consulting for routine care in the latter half of 2020 from across the United States (US) and 5 European countries (EU5): France, Germany, Italy, Spain, and the United Kingdom (UK). In addition to a physician screener and physician survey, physicians were asked to complete patient record forms on patient characteristics and treatment; these same patients were invited to fill out a voluntary patient self-completion questionnaire. Data reported in this study were collected at the time of the survey (e.g. demographic information) and previous recorded medical history (e.g. duration of treatment); although some data are related to specific time points (e.g. previous month, previous 12 months), there were no follow-up data collected in this study (Supplementary Fig. 1).

Eligible patients for this study were aged ≥ 12 years with a biopsy-confirmed physician diagnosis of EoE, an esophageal count of ≥ 15 eosinophils/high-power field (eos/hpf), and a current prescribed treatment for EoE. Patients were eligible for inclusion in this study regardless of the amount of time for which they had physician-diagnosed EoE.

### Assessments and statistical analyses

Assessments in this study included those for patient demographics, disease history, clinical characteristics, treatment pathways, patient symptom burden, and HRQOL measures (EuroQol 5-dimension 3-level [EQ-5D-3 L] assessment). Data for this study are summarized descriptively. Continuous variables are summarized by means, standard deviation, and medians (interquartile range and range). Categorical variables are reported as counts and percentages.

### Study conduct and ethics

The study was conducted according to standard operating procedures. Research was conducted as a survey in accordance with the amended Declaration of Helsinki, adhering to the ICC/ESOMAR International Code on Market, Opinion and Social Research and Data Analytics, the international code on observational research, and HIPAA (Health Insurance Portability and Accountability Act of 1996) guidelines. The survey was submitted to a central international review board (Western Institutional Review Board) and found to be exempt from ethics requirements on 23 July 2020 (Study Number: 1-1328144-1).

### Human ethics and consent to participate declarations

Not applicable.

## Results

### Patient demographics and clinical characteristics

This study focused on an overall population of patients with EoE, as well as a subgroup of those patients who had dysphagia despite treatment, as indicated by the diagnosing physician. Demographics and clinical characteristics were physician-reported, and HRQOL measures were patient-reported; the sources of all other data are indicated as follows with the associated data.

**Fig. 1:**
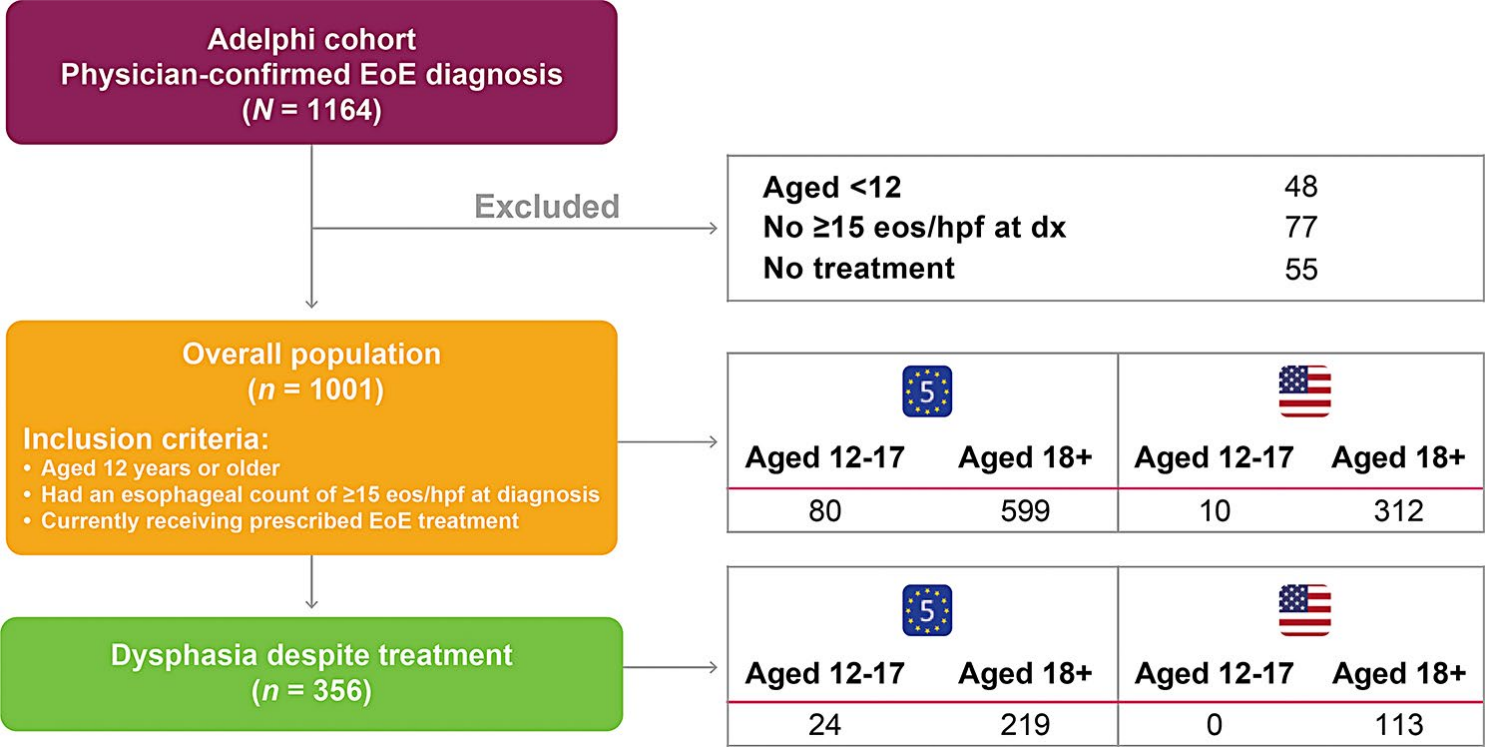
Patient population flowchart. EoE, eosinophilic esophagitis; eos/hpf, eosinophils per high-power field

Overall, there were 1001 patients included in this study, of which 356 (36%) had dysphagia despite treatment (Fig. 1). Demographics were generally similar between the overall and dysphagia-despite-treatment populations (Table 1). In the overall population, the mean (SD) age was 36.4 (14.58) years. Most patients were male (66%) and working full-time (58%), without caregiver help (90%). Clinical characteristics were also similar between the two patient populations (Table 2). The most common comorbidities among patients in the overall population were allergic rhinitis (*n* = 242, 24%) and asthma (*n* = 238, 24%). There were some differences in clinical characteristics in the overall and dysphagia-despite-treatment populations: 58% (*n* = 84/145) and 65% (*n* = 33/51) of patients, respectively, had ≥ 15 eos/hpf at the most recent endoscopy with biopsy and the current severity of EoE was mild in more patients in the overall group (*n* = 693, 69% vs. *n* = 171, 48%).

**Table 1:** Baseline demographics

Characteristic	Overall (*N* = 1001)	Dysphagia despite treatment (*N* = 356)
Age at screening (years), mean (SD)	36.4 (14.58)	36.8 (14.49)
Male sex, *n* (%)	657 (66)	236 (66)
BMI (kg/m^2^), mean (SD)	24.3 (3.63)	24.5 (3.56)
Employment status, *n* (%)	(*N* = 973)	(*N* = 341)
Working full-time	563 (58)	193 (57)
Working part-time	78 (8)	29 (9)
On long-term sick leave	7 (1)	3 (1)
Homemaker	48 (5)	15 (4)
Student	207 (21)	67 (20)
Retired	43 (4)	16 (5)
Unemployed	27 (3)	18 (5)
On long-term leave/homemaker/retired/unemployed owing to EoE, *n* (%)	(*N* = 121)	(*N* = 50)
Yes	5 (4)	3 (6)
No	116 (96)	47 (94)
Caregiver status, *n* (%)		
Yes	91 (10)	28 (8)
No	858 (90)	309 (92)

BMI, body mass index

**Table 2:** Clinical characteristics

Characteristic	Overall (*N* = 1001)	Dysphagia despite treatment (*N* = 356)
**Comorbidities in ≥ 5% of patients, ** ***n (%)***		
Asthma	238 (24)	83 (23)
Mild	133 (56)	49 (59)
Moderate	93 (39)	31 (37)
Severe	12 (5)	3 (4)
Allergic rhinitis	242 (24)	75 (21)
Anxiety	141 (14)	68 (19)
Atopic dermatitis	78 (8)	29 (8)
Depression	57 (6)	29 (8)
None of the above	376 (38)	123 (35)
Oral allergy syndrome	62 (6)	20 (6)
Peanut allergy	61 (6)	29 (8)
**Peak eos/hpf at most recent endoscopy with biopsy,** ***n*** **(%)**	(*N* = 145)	(*N* = 51)
< 6	23 (16)	4 (8)
6–15	38 (26)	14 (27)
≥ 15	84 (58)	33 (65)
**Current EoE severity**, ***n *(%)**		
Mild	693 (69)	171 (48)
Moderate	269 (27)	156 (44)
Severe	39 (4)	29 (8)

EoE, eosinophilic esophagitis; eos/hpf, eosinophils per high-powered field

### Patient disease and treatment history

**Fig. 2:**
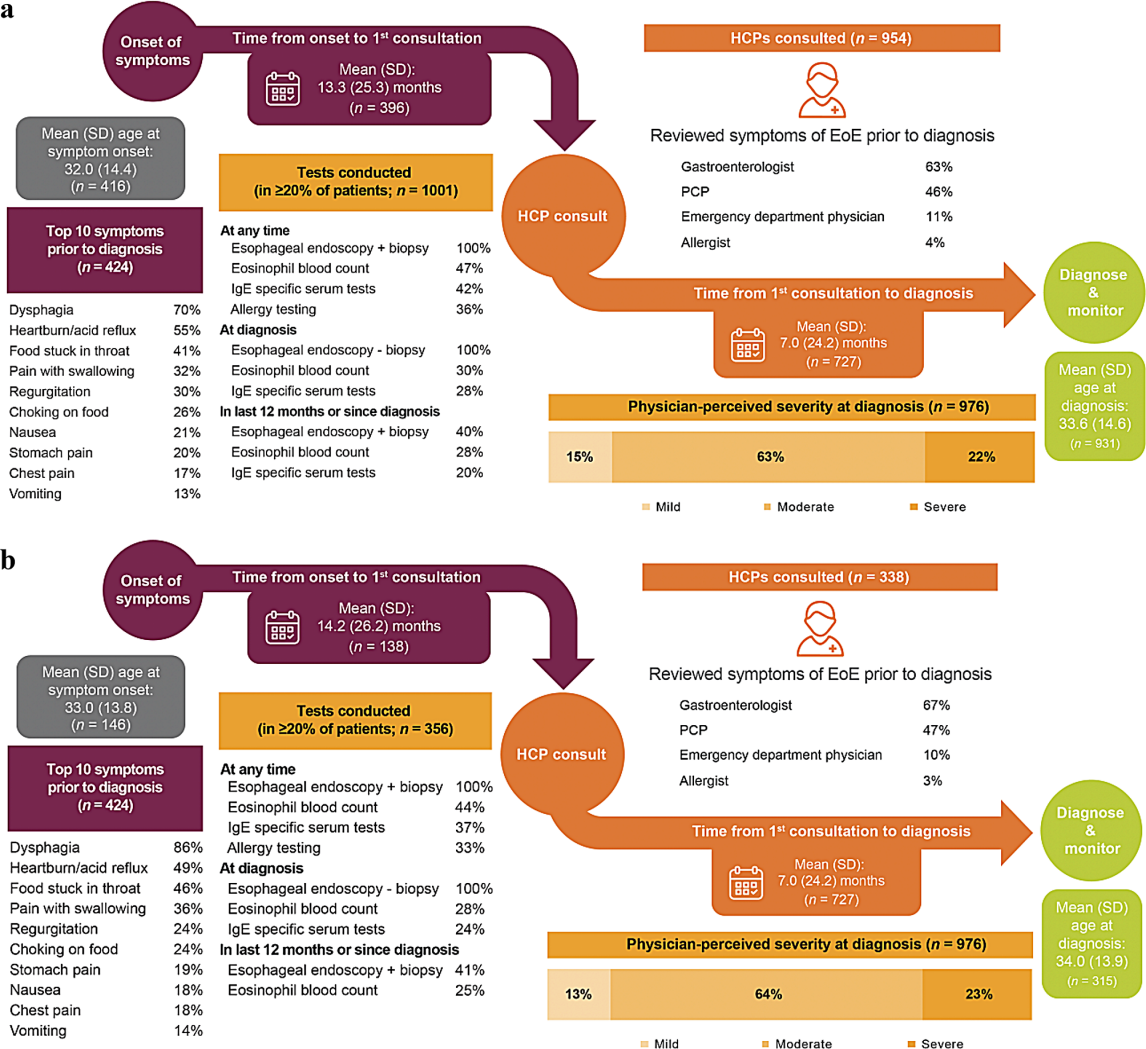
Patient journey from the onset of EoE symptoms to diagnosis (**a**) overall and (**b**) in patients with dysphagia despite treatment. EoE, eosinophilic esophagitis; HCP, health care provider; PCP, primary care provider

Patient disease history was similar in the overall and dysphagia-despite-treatment populations, respectively, with a few exceptions (Fig. 2). The most common patient-reported symptoms (*N* = 424 and *N* = 148) prior to diagnosis were dysphagia (70% and 86%) and heartburn/acid reflux (55% and 49%). The most common physician-reported symptoms (*N* = 972 and *N* = 341) were dysphagia (75% and 91%), food impaction (46% and 52%), reflux (45% and 40%), heartburn (32% and 25%), regurgitation (26% and 24%), choking on food (24% and 21%), and chest pain (22% and 24%), indicating differences between patient and physician reports. In the overall population, the mean (SD) time since the onset of symptoms to the first consultation was > 1 year (13.3 [25.26] months); patients were diagnosed on average within 1 year after the first consultation (mean [SD]: 7.0 [24.2] months), at which time 22% of patients had severe EoE. Symptoms were usually reviewed by a gastroenterologist (63%) prior to diagnosis, followed by primary care physicians (46%) and emergency department physicians (11%). Patient disease history was similar in populations categorized by region (US and EU5; Supplementary Table 1).

Treatment history did not differ between the overall and dysphagia-despite-treatment populations (Table 3). In the overall population, the most common current treatments (physician-reported) were proton pump inhibitors (83%) and topical corticosteroids (51%), with approximately 2 medications prescribed per patient. Most patients were very compliant or fully compliant with their medication treatment plan (patient-reported; 75%), and the main reason for noncompliance was forgetting to take the medication (58%). Similar proportions in both populations have had to adhere to an elimination diet (patient-reported), with most patients at least moderately compliant with their elimination diet. Additionally, in the overall population, 20% (196/991) of patients underwent dilation owing to EoE (physician-reported); the mean (SD) number of dilations since diagnosis was 1.7 (1.31; *N* = 185).

**Table 3:** Treatment history

Characteristic, *n* (%)	Overall (*N* = 1001)	Dysphagia despite treatment (*N* = 356)
**Physician-reported**		
Current prescribed pharmacological treatments		
Topical corticosteroids	507 (51)	212 (60)
Oral corticosteroids	86 (9)	30 (8)
Systemic corticosteroids	2 (< 1)	2 (1)
Other corticosteroids – not specified	115 (11)	46 (13)
Proton pump inhibitor	831 (83)	299 (84)
Biologic therapy	32 (3)	7 (2)
Antihistamine	152 (15)	48 (13)
Latency reversal agents	66 (7)	18 (5)
Number of treatments currently prescribed, mean (SD)	1.8 (0.85)	1.9 (0.81)
Details of dilations due to EoE		
Undergone dilation?	(*N* = 991)	(*N* = 349)
Yes	196 (30)	90 (26)
No	795 (70)	259 (74)
Number of dilations since diagnosis	(*N* = 185)	(*N* = 84)
Mean (SD)	1.5 (0.83)	1.8 (1.72)
**Patient-reported**		
Compliance with prescribed treatment	(*N* = 417)	(*N* = 144)
Fully compliant	184 (44)	52 (36)
Very compliant	128 (31)	46 (32)
Moderately compliant	92 (22)	36 (25)
Slightly compliant	12 (3)	9 (6)
Not at all compliant	1 (< 1)	1 (1)
Reasons for lack of compliance	(*N* = 204)	(*N* = 79)
I don’t think medication is needed for my EoE	15 (7)	8 (10)
I have concerns or fears about taking steroids	42 (21)	16 (20)
I only need to take medication when symptoms get worse and/or when at risk of getting worse	33 (16)	12 (15)
I do not like to be reliant on my medication	66 (32)	29 (37)
I do not feel instant results	30 (15)	15 (19)
Taking medication interferes with my lifestyle	22 (11)	11 (14)
My EoE medication is not a high priority compared with other medications	8 (4)	6 (8)
The number of times I have to take medication is not convenient	12 (6)	7 (9)
I forget to take my medication	118 (58)	44 (56)
I have experienced side effects	7 (3)	4 (5)
I am concerned about the long-term safety of treatments	32 (16)	15 (19)
The cost of the medication is too high	13 (6)	8 (10)
Other	1 (< 1)	0
**Details on diet**		
Foods eliminated	(*N* = 416)	(*N* = 142)
Yes, currently	124 (30)	124 (37)
Not currently but have in the past	107 (26)	107 (18)
No, never	185 (44)	185 (45)
Compliance with elimination diet	(*N* = 223)	(*N* = 76)
Fully	43 (19)	21 (28)
Very	72 (32)	10 (26)
Moderately	87 (39)	26 (34)
Slightly	18 (8)	8 (11)
Not at all	3 (1)	1 (1)

EoE, eosinophilic esophagitis

### Patient symptom burden

**Fig. 3:**
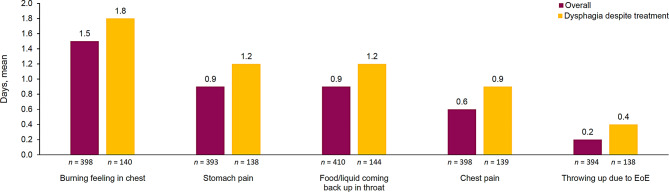
Seven-day symptom burden

On average, patients in the dysphagia-despite-treatment population reported slightly more days per week with symptoms versus the overall population (Fig. 3), with patients experiencing a range of different symptoms, from mild to severe. In the dysphagia-despite-treatment and overall populations, respectively, patients experienced an average of 1.8 versus 1.5 days with a burning feeling in the chest; 1.2 versus 0.9 days either with food or liquid coming back up into the throat or with stomach pain; and ≤ 1 day of chest pain or throwing up due to EoE in both populations. Similar results were observed by breakdown of US and EU5 populations (Supplementary Fig. 2); however, the mean number of days in which patients experienced chest pain and stomach pain were, on average, notably larger in the EU5 populations versus the US populations.

**Fig. 4:**
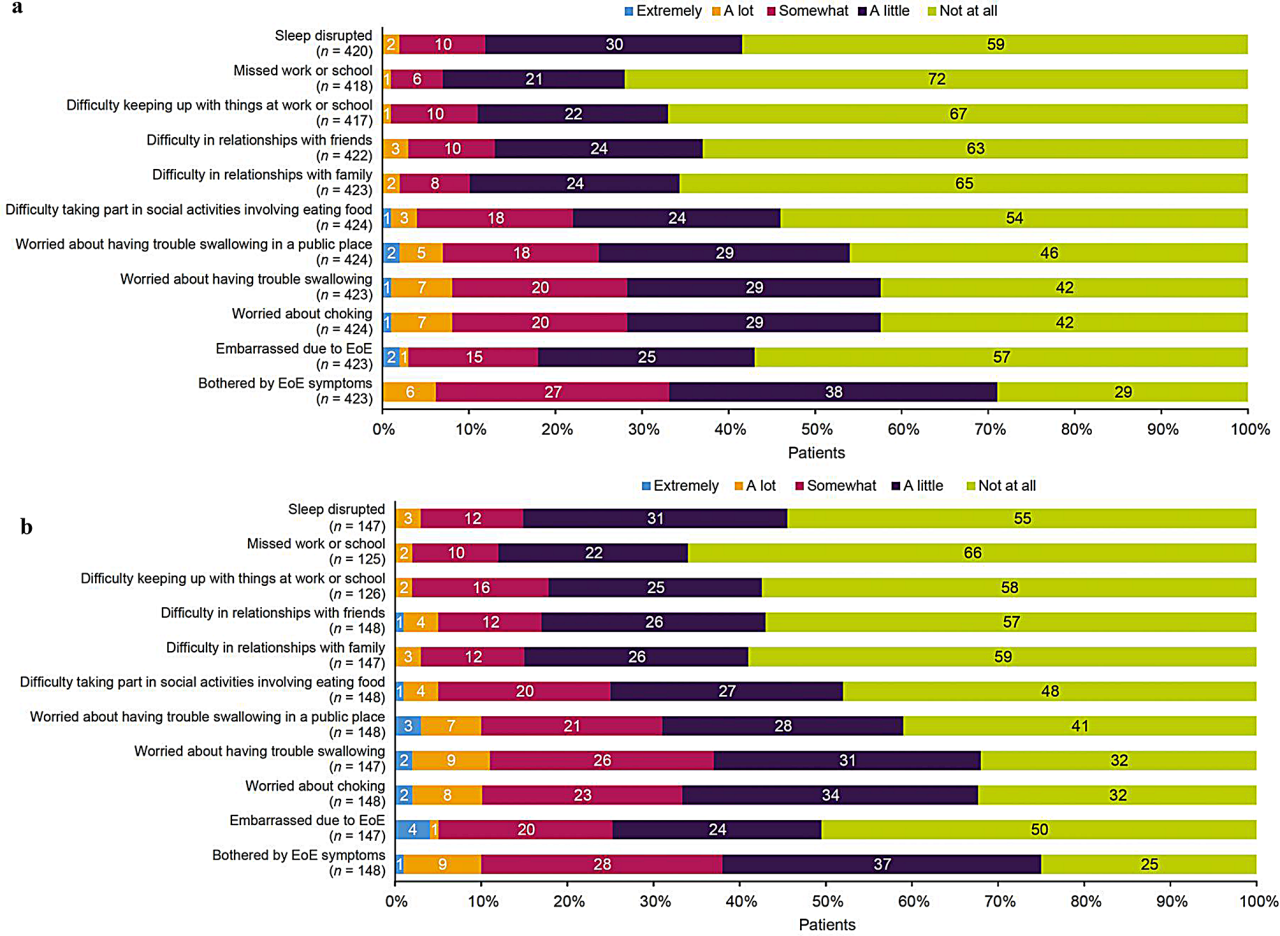
Seven-day impact on activities of daily living (**a**) overall and (**b**) in patients with dysphagia despite treatment.^a^ EoE, eosinophilic esophagitis. ^a^Numbers may not sum to 100% owing to rounding

Patients reported higher 7-day impacts on activities of daily living in the dysphagia-despite-treatment versus the overall population (Fig. 4), with patients in both populations experiencing impacts on their sleep, eating habits, and social lives at least a little or a lot each week. In the overall versus dysphagia-despite-treatment populations, respectively, patients reported being at least a little worried about choking (58% vs. 68%); at least a little worried about having trouble swallowing (58% vs. 68%), in a public place as well (54% vs. 59%); and at least a little difficulty taking part in social activities involving food (46% vs. 52%). Additionally, 37% versus 43% of patients in the overall versus dysphagia-despite-treatment populations, respectively, reported at least a little difficulty in relationships with friends, and 35% versus 41% reported at least a little difficulty in relationships with family. These trends were similar in the breakdown by US and EU5 populations (Supplementary Fig. 3 and Supplementary Fig. 4, respectively).

There were some differences in the most troublesome symptoms (physician-reported) between the two populations studied. In the overall (*N* = 528) and dysphagia-despite-treatment (*N* = 281) populations, respectively, the top troublesome symptoms were dysphagia (23% vs. 43%), food impaction (17% vs. 23%), reflux (13% vs. 5%), and heartburn (11% vs. 6%; Table 4). The mean (SD) patient EQ-5D-3 L utility scores were 0.929 (0.11; *N* = 390) versus 0.912 (0.13; *N* = 139) in the overall versus dysphagia-despite-treatment populations, respectively. The mean (SD) EQ-5D visual analog scale (VAS) scores, however, were similar in the overall population versus the dysphagia-despite-treatment population, respectively (83.3 [12.3], *N* = 377 vs. 81.2 [13.6], *N* = 135). The symptom burden data breakdown by US and EU5 populations can be found in Supplementary Table 2.

**Table 4:** Symptom burden

Characteristic, *n* (%)	Overall (*N* = 1001)	Dysphagia despite treatment (*N* = 356)
**Physician-reported**		
Top 10 most troublesome symptoms to patient, *n* (%)	*N* = 528	*N* = 281
Dysphagia	121 (23)	121 (43)
Food impaction	89 (17)	65 (23)
Reflux	71 (13)	15 (5)
Heartburn	58 (11)	17 (6)
Choking on food	36 (7)	16 (6)
Food-related anxiety (e.g. fear of impaction)	34 (6)	14 (5)
Regurgitation	23 (4)	8 (3)
Stomach pain	15 (3)	2 (1)
Nausea	8 (2)	3 (1)
Vomiting	6 (1)	0
**Patient-reported**		
EQ-5D VAS score, mean (SD)	*N* = 377	*N* = 135
	83.3 (12.26)	81.2 (13.59)
EQ-5D-3 L score, mean (SD)	*N* = 390	*N* = 139
	0.929 (0.11)	0.912 (0.13)

EoE, eosinophilic esophagitis; EQ-5D VAS, EuroQol 5-dimension visual analog scale; EQ-5D-3 L, EuroQol 5-dimension 3-level version

**Fig. 5:**
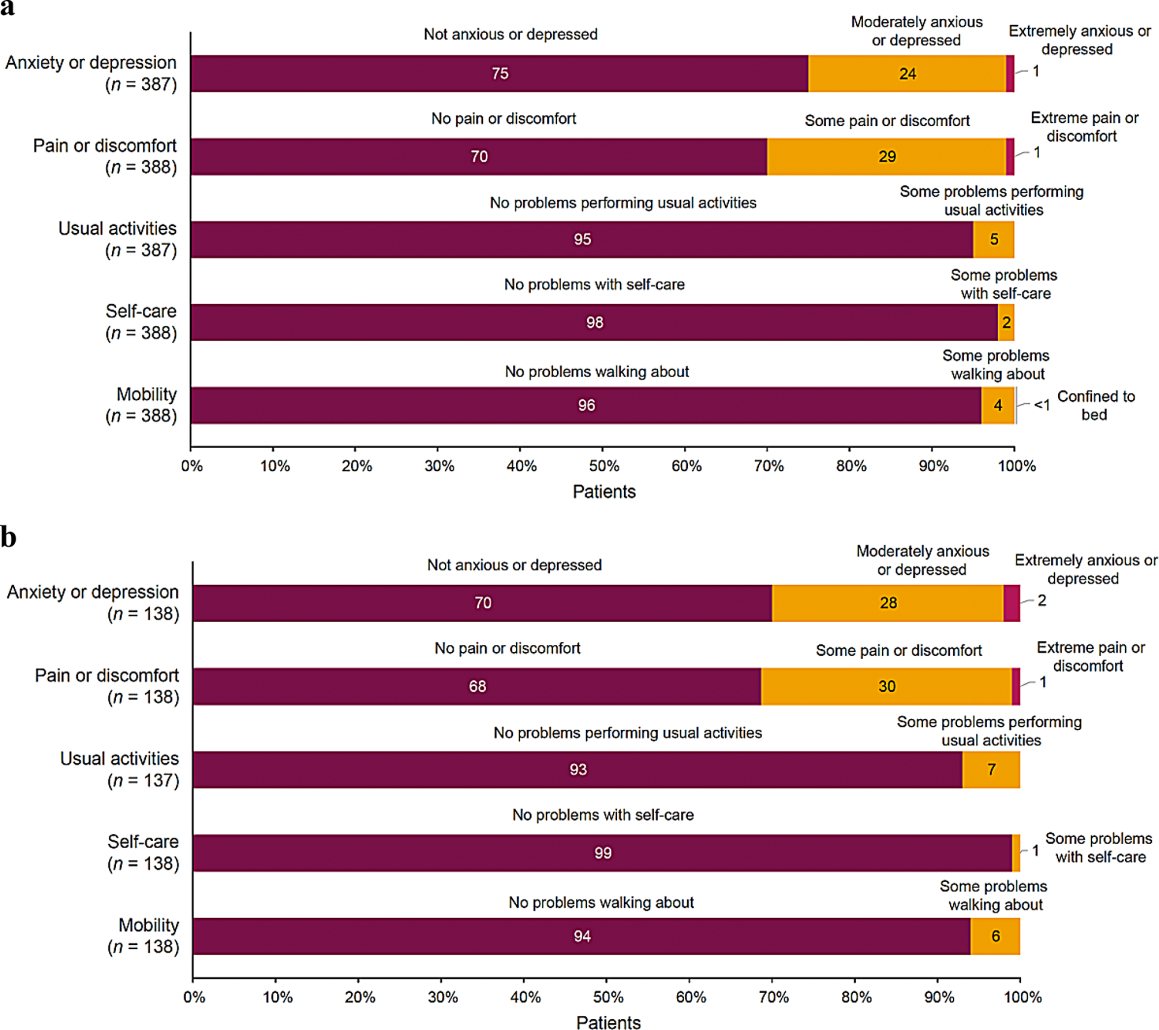
EQ-5D-3L utility domain responses (**a**) overall and (**b**) in patients with dysphagia despite treatment.^a^ EQ-5D-3L, EuroQol 5-dimension 3-level. ^a^Numbers may not sum to 100% owing to rounding

Outcomes from the EQ-5D-3 L utility score indicate slightly higher anxiety or depression in the dysphagia-despite-treatment population versus the overall population, with 30% versus 25% reporting at least moderate anxiety or depression (Fig. 5). Of note, 30% and 32% of patients in the overall and dysphagia-despite-treatment populations, respectively, reported at least moderate pain or discomfort. Outcomes by breakdown into US and EU5 populations generally follow similar trends as well (Supplementary Fig. 5 and Supplementary Fig. 6, respectively); however, greater proportions of patients in the EU5 experienced pain or discomfort and had at least some problems with self-care in the overall population versus the dysphagia-despite-treatment population.

## Discussion

This real-world study evaluated clinical characteristics, treatment history, and symptom burden in patients with EoE. Although the average time between the first healthcare provider consultation to EoE diagnosis was approximately half the time between the onset of symptoms to the first healthcare provider consultation, the high standard deviation in both of the populations we evaluated indicates significant wait time. In addition, by the time patients were diagnosed with EoE, a proportion of patients may have progressed to severe disease. Treatment history data, which were collected prior to the approval of dupilumab for EoE [13], were similar between both populations. Regardless of the congruent treatment histories, results from this study further demonstrate that EoE substantially impacts patients’ HRQOL and activities of daily living, which includes negative effects on their social, mental, and emotional well-being. Patients with dysphagia despite treatment experience higher symptom and HRQOL burden with dysphagia itself and anxiety or depression per EQ-5D-3 L responses, further underscoring the urgent need for novel, targeted treatment for patients with EoE. Moreover, because there were few notable differences in the overall versus dysphagia-despite-treatment populations, treatment of EoE patients warrants close follow-up and open discussions around symptoms to ensure the disease is well-managed.

In addition to the higher symptom burden experienced among patients with dysphagia despite treatment, outcomes from this study show that there is some discordance between patients and physicians regarding what each of them reports as being burdensome or worrisome. The most troublesome symptoms per physicians were dysphagia and food impaction; however, for patients, the most troublesome symptoms were food/liquid coming back up in the throat, stomach pain, and a burning feeling in the chest, and more than 50% of patients reported being worried about trouble when swallowing or choking. These differences reflect physicians’ lack of awareness of patients’ fears and how they might affect their lives.

Despite this discrepancy in awareness of patients’ fears, the current patient-reported assessment does not capture these anxieties. For example, the high EQ-5D-3 L VAS scores (indicating good health) and similar EQ-5D-3 L outcomes on usual activity, self-care, and mobility in both the overall and dysphagia-despite-treatment populations indicate that patients in both of these cohorts could lead a normal life; however, many impacts are not reflected in the current assessments. Social media studies also suggest a remaining unmet need for accurate sources of information about EoE [18, 19]. Although patients affected by chronic illnesses may often consult social media and patient-to-patient forums, there remains an unmet need for reliable information, owing to the lack of physician-verified claims and data. Given that EoE was first reported in the late 1960s [21] to 1970s [22, 23], few qualitative studies have been conducted that assess patient well-being through HRQOL [15–17], and outlets like social media may offer a wealth of evidence-based knowledge outside of the clinical setting that provides valuable insight into how patients actually perceive their EoE. Patients with EoE often have anxiety about eating owing to fear of choking, with the added psychosocial impact of not being able to eat normally and the resulting anxiety and/or embarrassment [12, 24]. Presenting symptoms may vary and make it challenging for a physician to diagnose properly. Supplementing physicians’ knowledge and understanding from the patient perspective is therefore crucial in order to make proper diagnoses and treatment recommendations. Patients may also view their disease as a series of distinct episodes that warrant management with as-needed strategies (e.g. chewing slowly and carefully); these behavioral modifications may therefore mask the impact of their symptoms when consulting their physicians, resulting in possible delays in diagnosis and treatment. While physicians are focused on diagnosing EoE to initiate pharmaceutical intervention as early as possible, per a recent qualitative analysis of three publicly accessible electronic health forums hosting EoE communities, many patients who recognized EoE as a chronic disease have voiced their preference for using dietary approaches over medications as a more permanent solution without reliance on lifelong medication use [19].

It is interesting to further note some slight differences in the overall versus dysphagia-despite-treatment poulations when stratified by location (i.e., US and EU5 countries). The types of tests conducted varied, with higher proportions of patients in the EU5 countries undergoing eosinophil blood count tests, IgE-specific serum tests, and allergy testing compared with patients in the US. The time from onset to first consultation was on average longer in the US compared with EU5 in both overall and dysphagia-despite-treatment populations. Higher patient-reported EQ-5D VAS scores and generally lower 7-day symptom burden were reported in the US compared with EU5 in both populations as well. Taken together, these results suggest that there are differences in healthcare system outputs coupled with variations in the EoE experience in the US compared with EU5 countries, which may impact how EoE is managed in these different regions.

Two strengths of this study are noteworthy: the study’s inclusion of large, robust numbers of patients and its objective and impartial nature. Both clinical and subjective variables are captured, with real-life patient-reported outcomes. Consistent methodology was used in all regions, allowing for true cross-country comparisons. Given that the study was descriptive in nature, there was no set hypothesis prior to data collection, providing flexibility in outcomes studied and potentially eliminating bias. A limitation is that this disease-specific study may ignore the presence of multiple chronic conditions. In addition, the patient surveys were voluntary: not all patients completed the questionnaire, and these data represent only the population of patients who did.

EoE is a multisymptomatic, chronic allergic inflammatory disease of the esophagus that can greatly hinder a patient’s overall well-being and quality of life, especially related to diet and eating, despite standard-of-care treatments. In addition to the need for novel treatments, there is also a substantial need for greater awareness of the condition, including the burdensome symptoms and disease journey faced by many patients with EoE, to encourage earlier diagnosis and closer management of the disease.

### Electronic supplementary material

Below is the link to the electronic supplementary material.

Supplementary Material 1

## Data Availability

This study was an analysis of secondary data accessed from a database of cross-sectional patient and physician survey data collected as part of the Adelphi Real World’s EoE Disease Specific Programme (DSP)^™^, and as such, the data that support the findings of this study are available from Adelphi. However, restrictions apply to the availability of these data, which were used under license for the current study, and so are not publicly available. Data are however available from the authors upon reasonable request and with permission of Adelphi.

## Abbreviations

EoE: Eosinophilic esophagitis
HRQoL: Health-related quality of life
EU: European Union
UK: United Kingdom
Eos/hpf: Eosinophils/high-power field
EQ-5D-3L: EuroQol 5-dimension 3-level
ICC/ESOMAR: International Code on Market/European Society for Opinion and Market Research
HIPAA: Health Insurance Portability and Accountability Act of 1996
SD: Standard deviation
VAS: Visual analog scale
