# Supplementary material for: Patient-reported symptoms and burden of eosinophilic esophagitis: evidence from real-world clinical practice
Source: BMC Gastroenterol. 2024 Aug 3;24:246. doi: 10.1186/s12876-024-03334-4 (PMC11297626; doi:10.1186/s12876-024-03334-4)

**Supplementary material**

**Patient-reported symptoms and burden of eosinophilic esophagitis: evidence from real-world clinical practice**

Xiao Xu,^1,2^* Justin Kwiatek,^2^ James Siddall,^3^ Eduardo Genofre,^4^ Heide Stirnadel-Farrant,^5^ Rohit Katial^2^

*****Corresponding author:

Xiao Xu, PhD

AstraZeneca

200 Orchard Ridge Drive

Gaithersburg, MD 20878 USA

Email: Xiao.Xu1@astrazeneca.com

Phone: +1 (301) 525-3669

Fax: +1 (302) 397-2810

**Contents:**

S1. Supplementary Table 1. Number of study participants by country

S2. Supplementary Table 2. Diagnostic history (US and EU5 breakdown)

S3. Supplementary Table 3. Physician-reported common treatments for patients with EoE

S4. Supplementary Table 4. Symptom burden (US and EU5 breakdown)

S5. Supplementary Fig. 1. Study design

S6. Supplementary Fig. 2. Seven-day symptom burden in the (a) US and (b) EU5 populations

S7. Supplementary Fig. 3. Seven-day impact on activities of daily living in the (a) overall and (b) dysphagia-despite-treatment populations (US population)

S8. Supplementary Fig. 4. Seven-day impact on activities of daily living in the (a) overall and (b) dysphagia-despite-treatment populations (EU5 population)

S9. Supplementary Fig. 5. EQ-5D-3L utility domain responses in the (a) overall and (b) dysphagia-despite-treatment populations (US population)

S10. Supplementary Fig. 6. EQ-5D-3L utility domain responses in the (a) overall and (b) dysphagia-despite-treatment populations (EU5 population)

**S1**

**Supplementary Table 1** Number of study participants by country

| **Total *N* = 1001** | **Overall, *N*** | **Dysphagia despite treatment, *N*** |
| --- | --- | --- |
| France | 122 | 42 |
| Germany | 135 | 49 |
| Italy | 132 | 45 |
| Spain | 160 | 44 |
| United Kingdom | 112 | 52 |
| United States | 318 | 109 |

**S2**

**Supplementary Table 2** Diagnostic history (US and EU5 breakdown)

| **Characteristic** | **US** | | | **EU5** | |
| --- | --- | --- | --- | --- | --- |
|  | **Overall**  ***N =* 322** | **Dysphagia despite treatment**  ***N =* 113** | | **Overall**  ***N =* 679** | **Dysphagia despite treatment**  ***N =* 243** |
| **Physician-reported** |  | |  |  |  |
|  | *N =* 214 | | *N =* 70 | *N =* 513 | *N =* 169 |
| Time from first consult to diagnosis, months, mean (SD) | 6.71 (24.2) | | 9.5 (33.8) | 7.16 (24.2) | 4.7 (8.67) |
|  | *N =* 295 | | *N =* 97 | *N =* 636 | *N =* 218 |
| Age at diagnosis, years, mean (SD) | 35.5 (13.13) | | 35.6 (12.87) | 32.7 (15.11) | 33.3 (14.31) |
| Reviewed symptoms prior to diagnosis, *n* (%) | *N =* 300 | | *N =* 109 | *N =* 654 | *N =* 235 |
| Gastroenterologist | 204 (68) | | 67 (65) | 400 (61) | 158 (67) |
| Primary care physician | 122 (41) | | 49 (48) | 321 (49) | 109 (46) |
| Emergency department physician | 24 (8) | | 8 (8) | 79 (12) | 27 (11) |
| Allergist | 15 (5) | | 4 (4) | 24 (4) | 5 (2) |
| Severity at diagnosis, *n* (%) | *N =* 308 | | *N =* 105 | *N =* 668 | *N =* 243 |
| Mild | 37 (12) | | 14 (13) | 113 (17) | 30 (12) |
| Moderate | 207 (67) | | 68 (65) | 406 (61) | 156 (64) |
| Severe | 64 (21) | | 23 (22) | 149 (22) | 57 (23) |
| Tests conducted in ≥20% of patients,^a^ *n* (%) |  | |  |  |  |
| At any time | *N =* 322 | | *N =* 113 | *N =* 679 | *N =* 243 |
| Esophageal endoscopy + biopsy | 322 (100) | | 113 (100) | 679 (100) | 243 (100) |
| Eosinophil blood count | 103 (32) | | 35 (31) | 367 (54) | 121 (50) |
| IgE-specific serum tests | 73 (23) | | 16 (14) | 352 (52) | 115 (47) |
| Allergy testing | 89 (28) | | 23 (20) | 267 (39) | 96 (40) |
| At diagnosis | *N =* 322 | | *N =* 113 | *N =* 679 | *N =* 243 |
| Esophageal endoscopy + biopsy | 322 (100) | | 113 (100) | 670 (99) | 243 (100) |
| Eosinophil blood count | 59 (18) | | 17 (15) | 245 (36) | 83 (34) |
| IgE-specific serum tests | 46 (14) | | 7 (6) | 237 (35) | 79 (33) |
| Allergy testing | 26 (8) | | 3 (3) | 145 (21) | 55 (23) |
| In the last 12 months or since diagnosis | *N =* 322 | | *N =* 113 | *N =* 679 | *N =* 243 |
| Esophageal endoscopy + biopsy | 100 (31) | | 38 (34) | 296 (44) | 107 (44) |
| Eosinophil blood count | 79 (25) | | 25 (22) | 206 (30) | 63 (26) |
| IgE-specific serum tests | 52 (16) | | 11 (10) | 149 (22) | 54 (22) |
| **Patient-reported** |  | |  |  |  |
|  | *N =* 175 | | *N =* 61 | *N =* 241 | *N =* 85 |
| Age at onset, years, mean (SD) | 33.1 (13.49) | | 33.2 (12.87) | 31.2 (15.01) | 32.8 (14.56) |
|  | *N =* 165 | | *N =* 59 | *N =* 231 | *N =* 79 |
| Time from onset to first consultation, months, mean (SD) | 15.2 (26.27) | | 17.3 (29.73) | 11.9 (24.47) | 12.0 (23.17) |
| Top 10 symptoms prior to diagnosis, *n* (%) | *N =* 177 | | *N =* 61 | *N =* 247 | *N =* 87 |
| Dysphagia | 108 (61) | | 48 (79) | 188 (76) | 80 (92) |
| Heartburn/acid reflux | 106 (60) | | 32 (52) | 126 (51) | 40 (46) |
| Food stuck in throat | 77 (44) | | 34 (56) | 97 (39) | 34 (39) |
| Choking on food | 61 (34) | | 19 (31) | 49 (20) | 17 (20) |
| Regurgitation | 48 (27) | | 16 (26) | 79 (32) | 20 (23) |
| Pain with swallowing | 39 (22) | | 20 (33) | 96 (39) | 34 (39) |
| Stomach pain | 24 (14) | | 11 (18) | 59 (24) | 17 (20) |
| Nausea | 22 (12) | | 6 (10) | 69 (28) | 21 (24) |
| Chest pain | 20 (11) | | 10 (16) | 50 (20) | 17 (20) |
| Vomiting | 14 (8) | | 5 (8) | 43 (17) | 15 (17) |

EU5, 5 European countries—France, Germany, Italy, Spain, and the United Kingdom; IgE, immunoglobulin E; SD, standard deviation.

^a^In any subgroup.

**S3**

**Supplementary Table 3** Physician-reported common treatments for patients with EoE

|  | **Overall, *N*** | **Dysphagia despite treatment, *N*** |
| --- | --- | --- |
| Topical corticosteroid | 507 | 212 (42%) |
| Oral corticosteroid | 86 | 30 (35%) |
| Systemic corticosteroid | 2 | 2 (100%) |
| Other corticosteroid, not specified | 115 | 46 (40%) |
| Proton pump inhibitor | 831 | 299 (36%) |
| Biologic therapy | 32 | 7 (22%) |
| Antihistamine | 152 | 48 (32%) |
| Leukotriene receptor antagonist | 66 | 18 (27%) |

S4

**Supplementary Table 4** Symptom burden (US and EU5 breakdown)

| **Characteristic** | **US** | | **EU5** | |
| --- | --- | --- | --- | --- |
|  | **Overall**  ***N =* 322** | **Dysphagia despite treatment**  ***N =* 113** | **Overall**  ***N =* 679** | **Dysphagia despite treatment**  ***N =* 243** |
| **Physician-reported** |  |  |  |  |
| Top 10 most troublesome symptoms to patient, *n* (%) | *N =* 166 | *N =* 91 | *N =* 362 | *N =* 190 |
| Dysphagia | 43 (26) | 43 (47) | 78 (22) | 78 (41) |
| Reflux | 29 (17) | 7 (8) | 42 (12) | 8 (4) |
| Heartburn | 24 (14) | 9 (10) | 34 (9) | 8 (4) |
| Food impaction | 21 (13) | 15 (16) | 68 (19) | 50 (26) |
| Choking on food | 19 (11) | 10 (11) | 17 (5) | 6 (3) |
| Regurgitation | 8 (5) | 3 (3) | 15 (4) | 5 (3) |
| Food-related anxiety (e.g., fear of impaction) | 5 (3) | 1 (1) | 29 (8) | 13 (7) |
| Stomach pain | 4 (2) | 1 (1) | 11 (3) | 1 (1) |
| Vomiting | 3 (2) | 0 | 3 (1) | 0 |
| Nausea | 1 (1) | 0 | 7 (2) | 3 (2) |
| **Patient-reported** |  |  |  |  |
|  | *N =* 167 | *N =* 61 | *N =* 210 | *N =* 74 |
| EQ-5D VAS score, mean (SD) | 86.5 (11.09) | 84.6 (11.87) | 80.8 (12.58) | 78.5 (14.37) |
|  | *N =* 169 | *N =* 61 | *N =* 221 | *N =* 78 |
| EQ-5D-3L score, mean (SD) | 0.944 (0.11) | 0.930 (0.11) | 0.918 (0.11) | 0.899 (0.14) |

EoE, eosinophilic esophagitis; EQ-5D VAS, EuroQol 5-dimension visual analog scale; EQ-5D-3L, EuroQol 5-dimension 3-level version; EU5, 5 European countries—France, Germany, Italy, Spain, and the United Kingdom; SD, standard deviation; US, United States.

**S5**

**Supplementary Fig. 1** Study design

HCP, health care provider; EoE, eosinophilic esophagitis; EQ-5D VAS, EuroQol 5-dimension visual analog scale; EQ-5D-3L, EuroQol 5-dimension 3-level version; Q3-4, quarters 3 to 4.


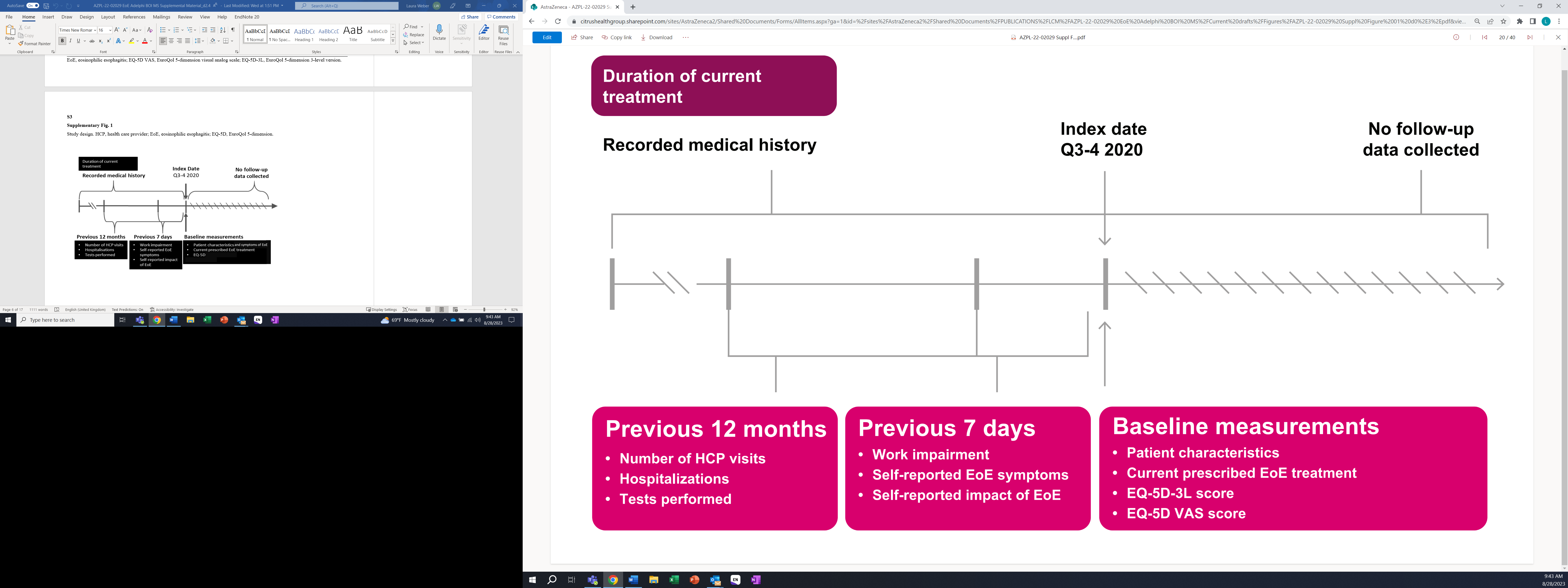


**S6**

**Supplementary Fig. 2** Seven-day symptom burden in the (a) US and (b) EU5 populations

EoE, eosinophilic esophagitis; EU5, 5 European countries—France, Germany, Italy, Spain, and the United Kingdom; US, United States.

**a**


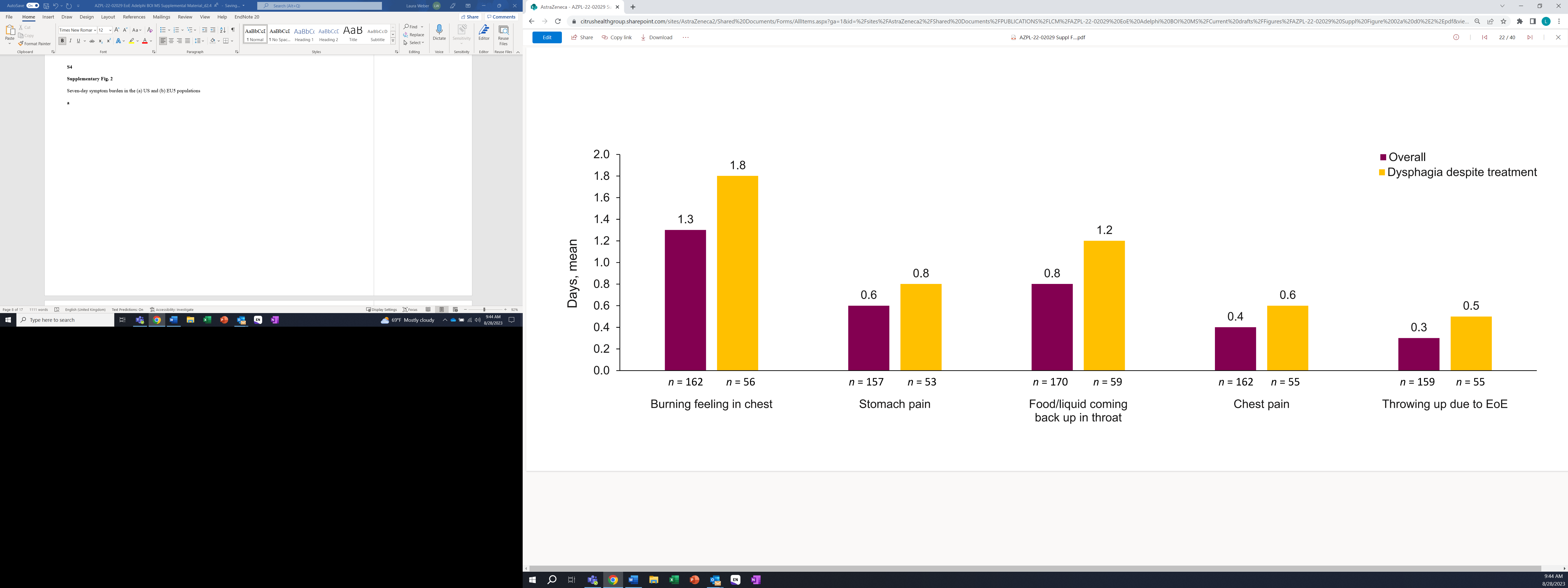


**b**


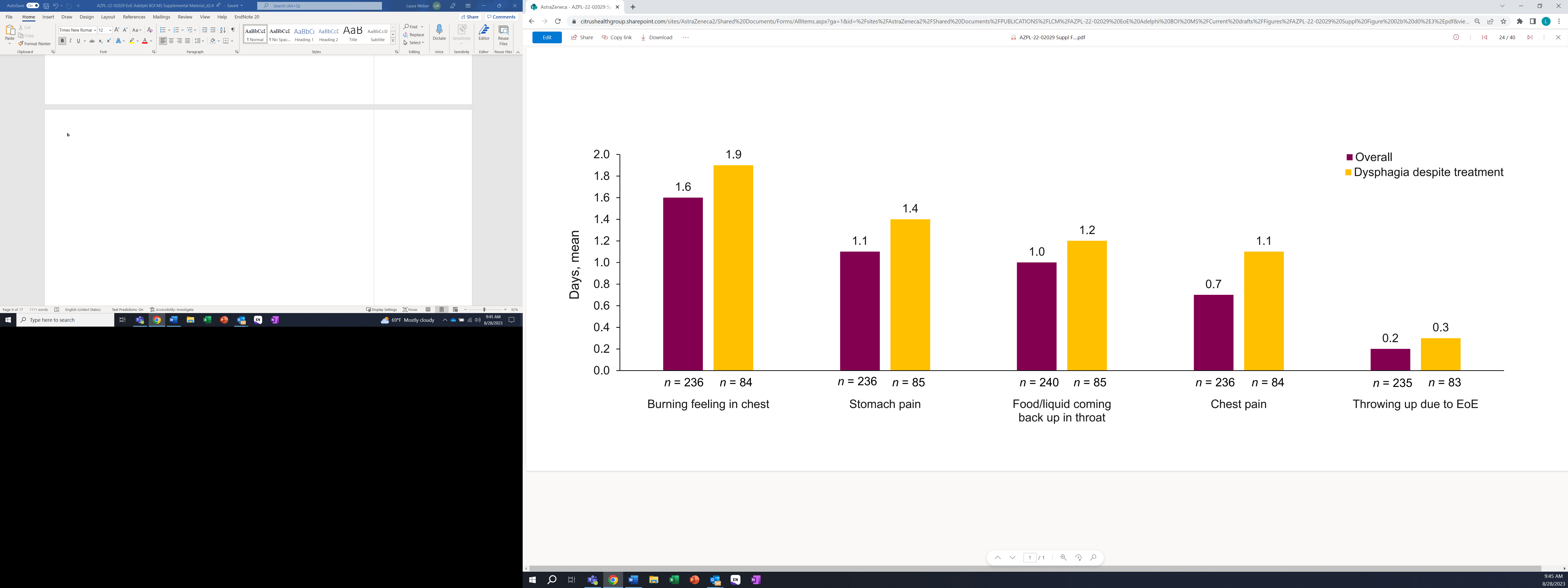


**S7**

**Supplementary Fig. 3** Seven-day impact on activities of daily living in the (a) overall and (b) dysphagia-despite-treatment populations (US population)^a^

EoE, eosinophilic esophagitis; US, United States.

^a^Numbers may not add up to 100% owing to rounding.

**a**


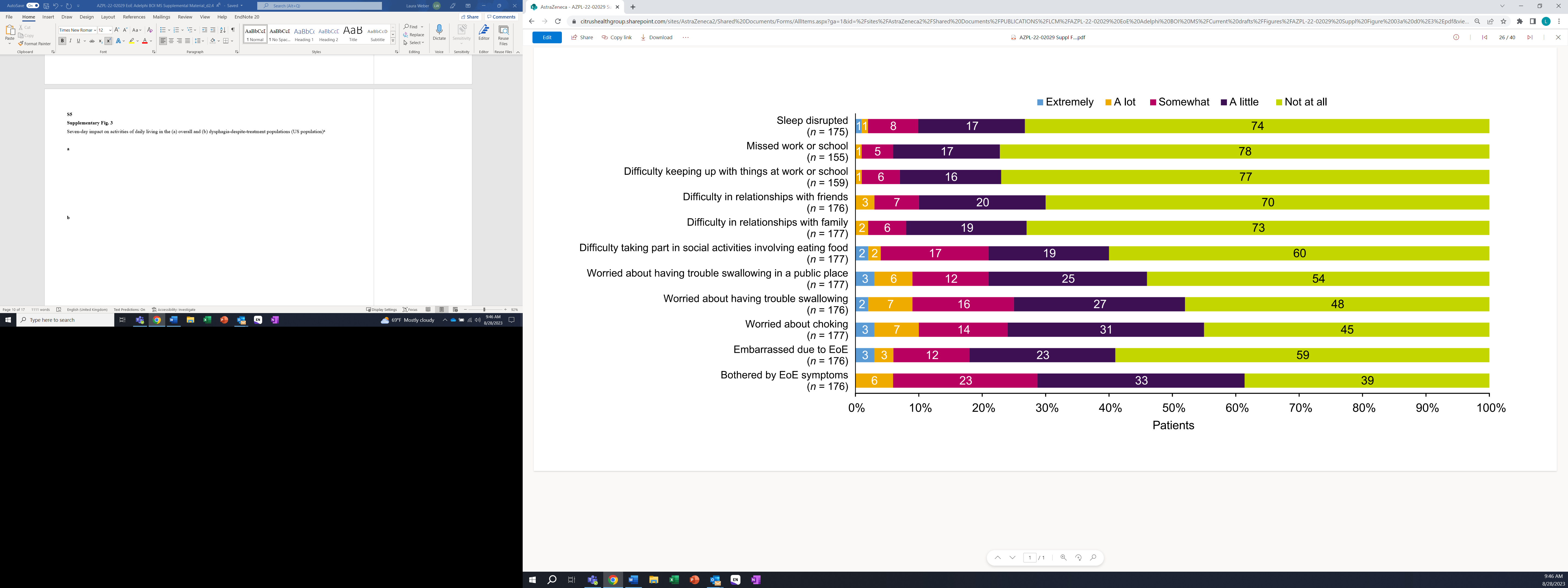


**b**


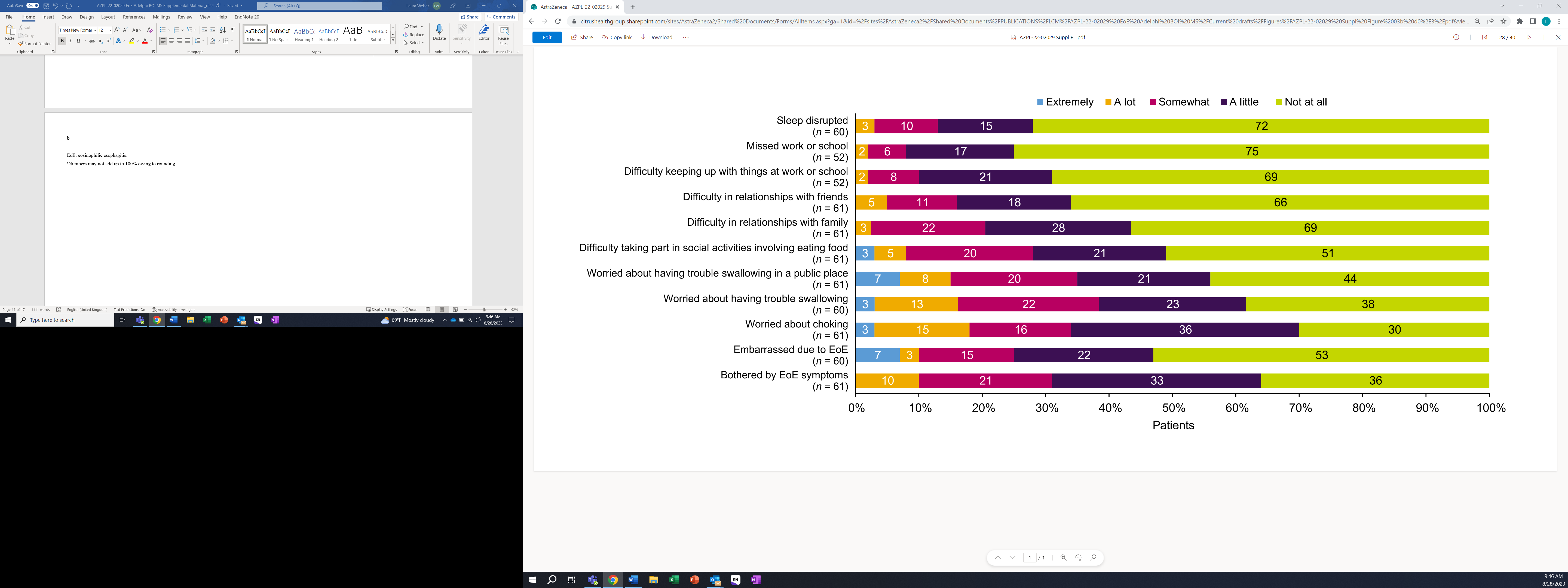


**S8**

**Supplementary Fig. 4** Seven-day impact on activities of daily living in the (a) overall and (b) dysphagia-despite-treatment populations (EU5 population)^a^

EoE, eosinophilic esophagitis; EU5, 5 European countries—France, Germany, Italy, Spain, and the United Kingdom.

^a^Numbers may not add up to 100% owing to rounding.

**a**


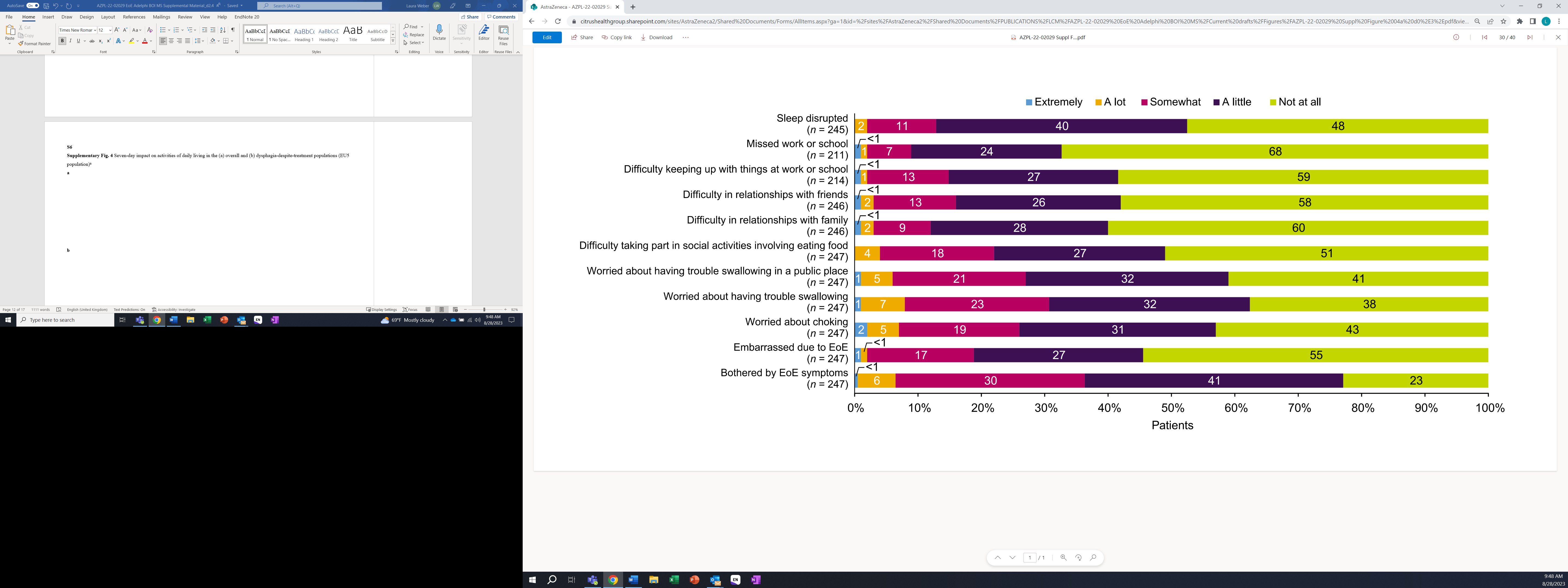


**b**


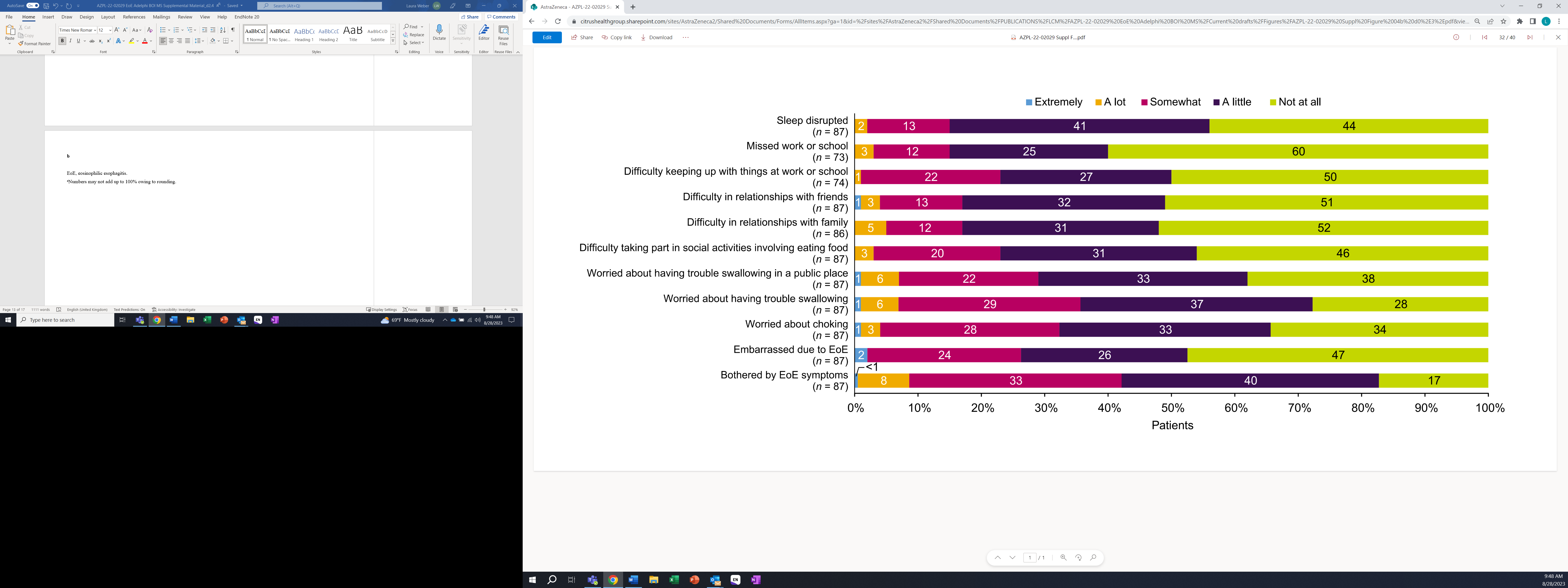


**S9**

**Supplementary Fig. 5** EQ-5D-3L utility domain responses in the (a) overall and (b) dysphagia-despite-treatment populations (US population)^a^

EQ-5D-3L, EuroQol 5-dimension 3-level version; US, United States.

^a^Numbers may not add up to 100% owing to rounding.

**a**


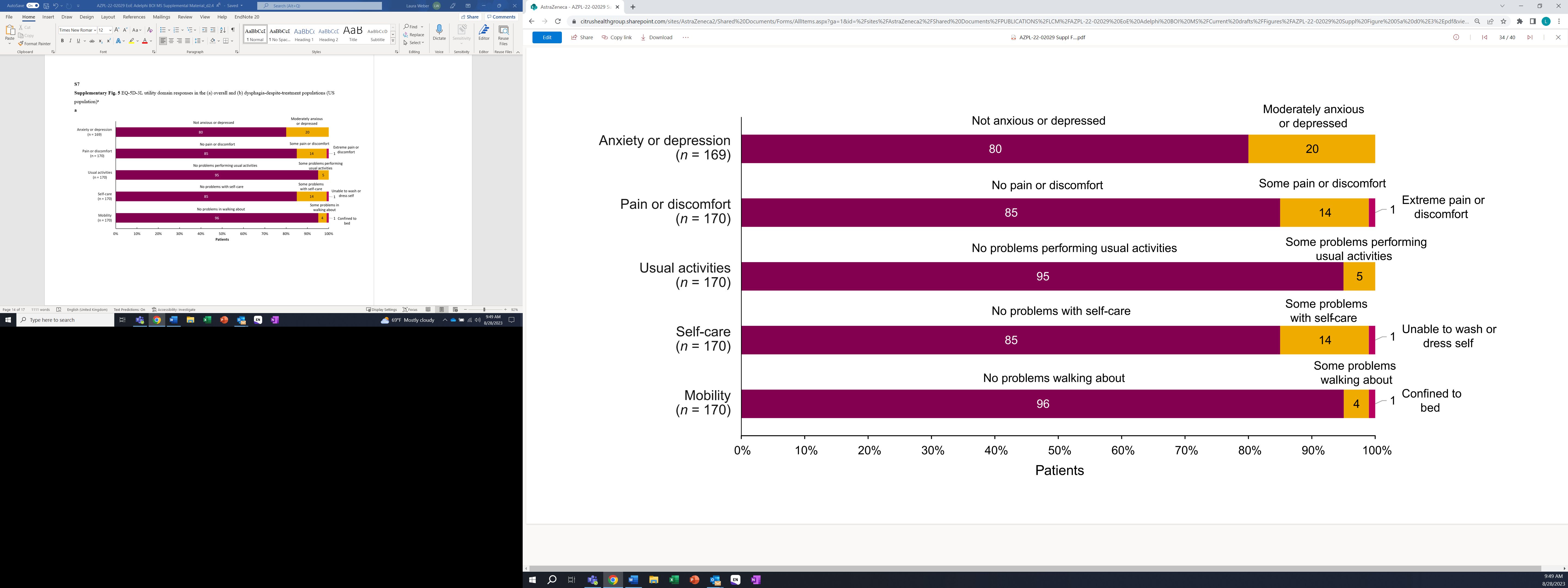


**b**


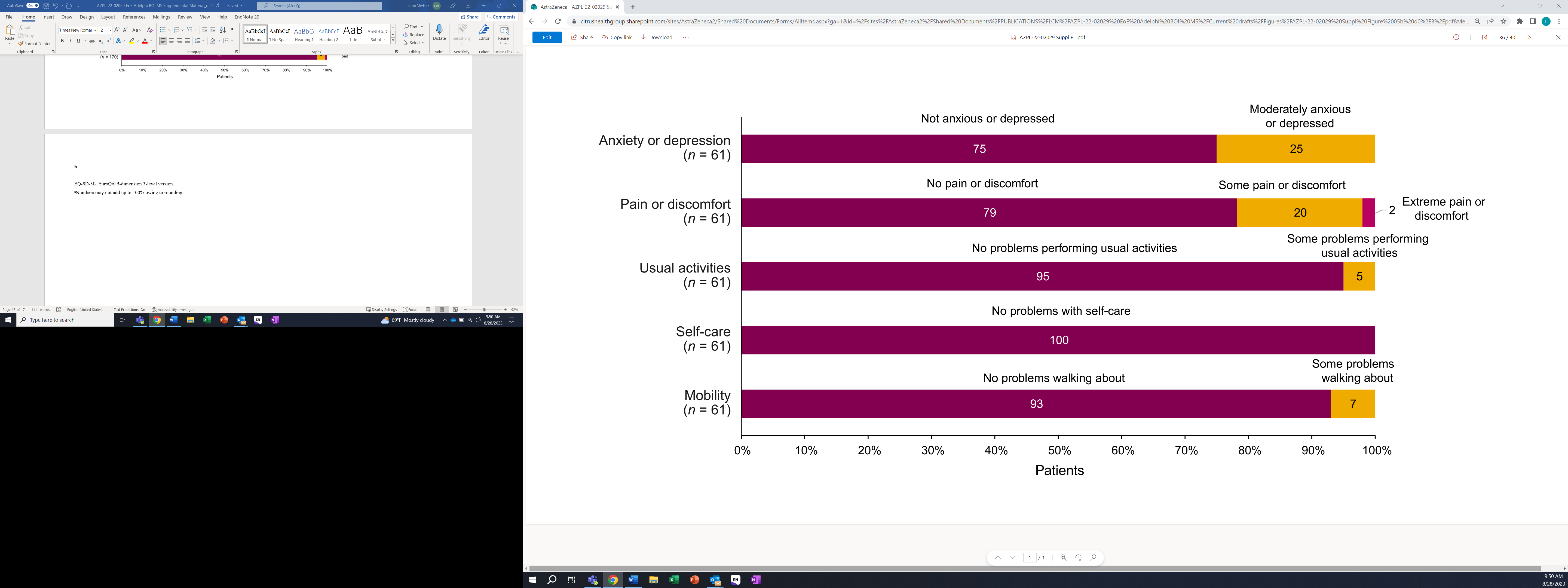


**S10**

**Supplementary Fig. 6** EQ-5D-3L utility domain responses in the (a) overall and (b) dysphagia-despite-treatment populations (EU5 population)^a^

EQ-5D-3L, EuroQol 5-dimension 3-level version; EU5, 5 European countries—France, Germany, Italy, Spain, and the United Kingdom.

^a^Numbers may not add up to 100% owing to rounding.

**a**


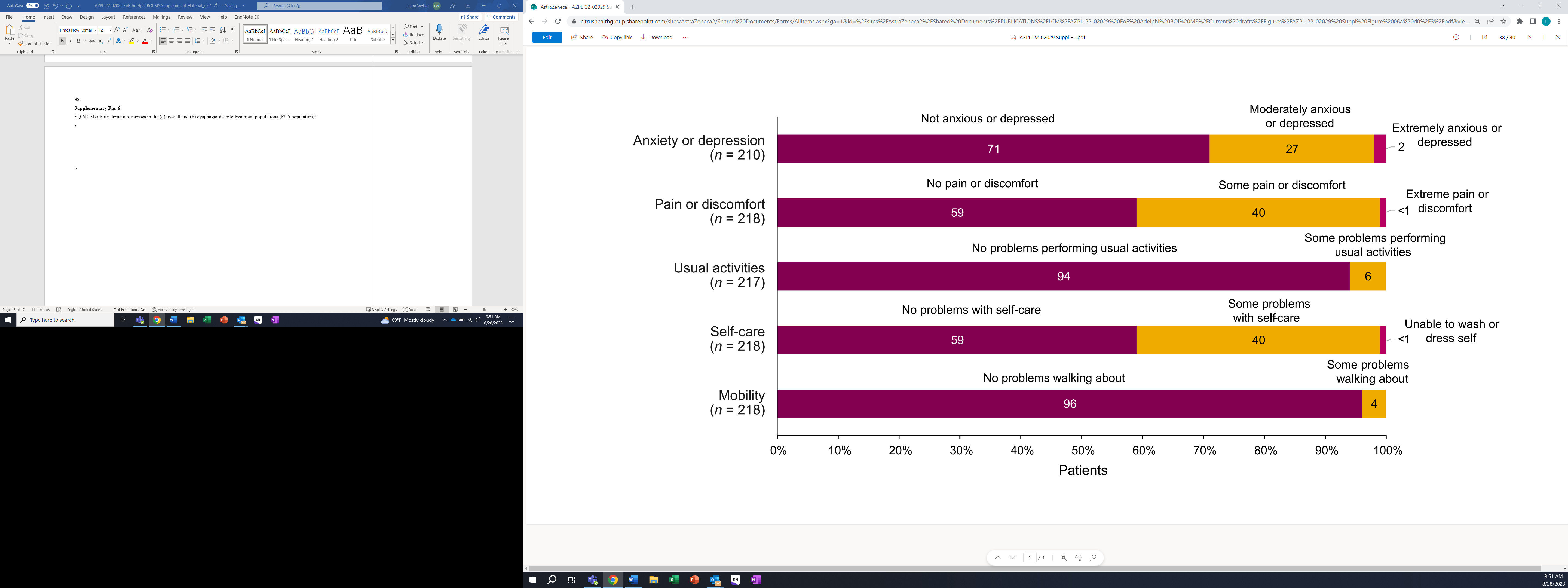


**b**


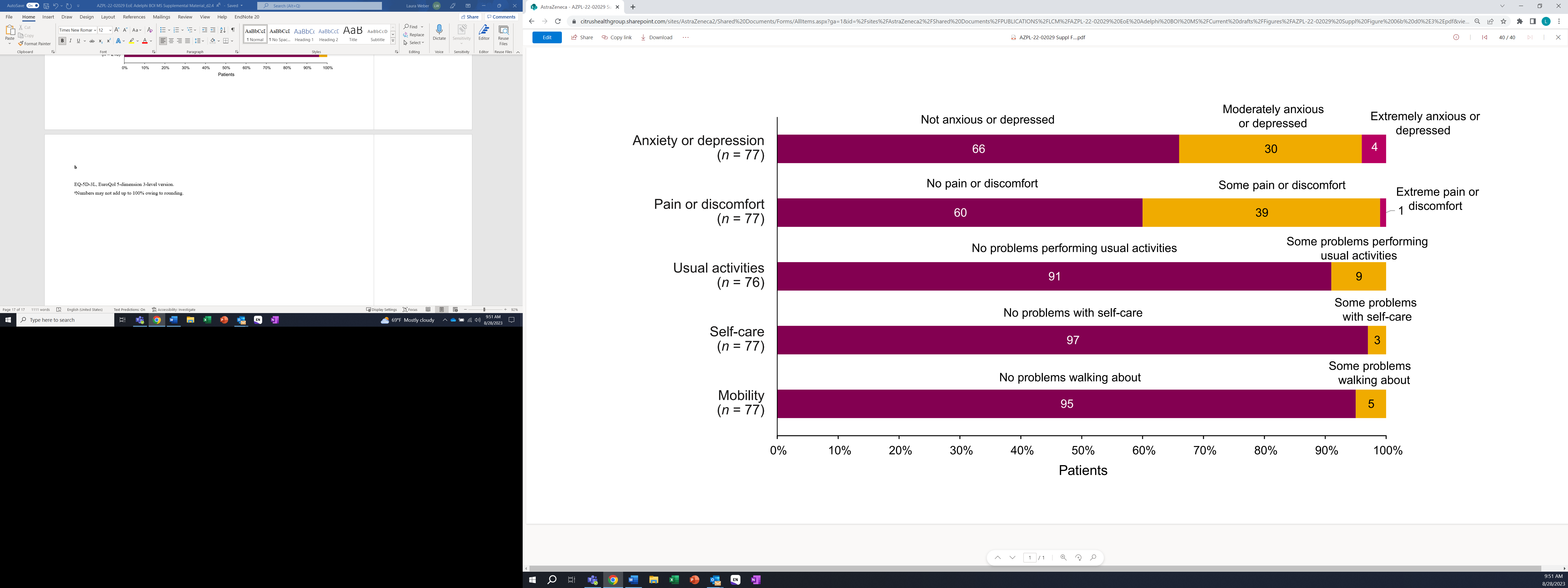

Supplement: Supplementary file 1 — Supplementary Material 1 [file 12876_2024_3334_MOESM1_ESM.docx]
